# Re-irradiating spinal column metastases using IMRT and VMAT with and without flattening filter - a treatment planning study

**DOI:** 10.1186/s13014-016-0603-0

**Published:** 2016-03-01

**Authors:** Barbara Dobler, Amine Khemissi, Tina Obermeier, Matthias G. Hautmann, Zaira Katsilieri, Oliver Kölbl

**Affiliations:** Department of Radiotherapy, Regensburg University Medical Center, Regensburg, Germany

## Abstract

**Background:**

The aim of this study was to investigate the potential of the flattening filter free (FFF) mode of a linear accelerator for intensity modulated radiation therapy (IMRT) and volumetric modulated arc therapy (VMAT) for patients with in-field recurrence of vertebral metastases.

**Methods:**

An Elekta Synergy Linac with Agility™ head is used to simulate the treatment of ten patients with locally recurrent spinal column metastases. Four plans were generated for each patient treating the vertebrae sparing the spinal cord: Dual arc VMAT and nine field step and shoot IMRT each with and without flattening filter. Plan quality was assessed considering target coverage and sparing of the spinal cord and normal tissue. All plans were verified by a 2D-ionisation-chamber-array, peripheral doses were measured and compared to calculations. Delivery times were measured and compared. The Wilcoxon test was used for statistical analysis with a significance level of 0.05.

**Results:**

Target coverage, homogeneity index and conformity index were comparable for both flat and flattening filter free beams. The volume of the spinal cord receiving the allowed maximum dose to keep the risk of radiation myelopathy at 0 % was at the same time significantly reduced to below the clinically relevant 1 ccm using FFF mode. In addition the mean dose deposited in the surrounding healthy tissue was significantly reduced in the FFF mode. All four techniques showed equally good gamma scores for plan verification. FFF plans required considerably more MU per fraction dose. Regardless of the large number of MU, out-of-field point dose was significantly lower for FFF plans, with an average reduction of 33 % and mean delivery time was significantly reduced by 22 % using FFF beams. When compared to IMRT FF, VMAT FFF offered even a reduction of 71 % in delivery time and 45 % in peripheral dose.

**Conclusions:**

FFF plans showed a significant improvement in sparing of normal tissue and the spinal cord, keeping target coverage and homogeneity comparable. In addition, delivery times were significantly reduced for FFF treatments, minimizing intrafractional motion as well as strain for the patient. Shortest delivery times were achieved using VMAT FFF. For radiotherapy of spinal column metastases VMAT FFF may therefore be considered the preferable treatment option for the combination of Elekta Synergy Linacs and Oncentra® External Beam v4.5 treatment planning system.

## Background

The flattening filter in the treatment head of a linear accelerator allows achieving a homogeneous dose profile for photon beams. In the past this was necessary for 2D radiation therapy planning and facilitated forward 3D radiation therapy planning. The use of flattening filters implies, however, also certain disadvantages: The flattening filter reduces photon fluence, leading to lower dose rates and prolonged beam-on times, and increases scatter dose produced in the treatment head [1–3]. Through the development of intensity modulated radiation therapy (IMRT) and the associated inverse treatment planning, a homogeneous beam profile is no longer necessary. The latest development in the technology of linear accelerators is therefore the opportunity to irradiate patients without a flattening filter in the beam path to increase dose rate and reduce beam-on times as well as out-of-field doses [1]. The physical properties of flattening filter free beams have been subject to a wide number of investigations during the last years [4–14]. Several treatment planning studies have been published to assess the clinical value of FFF beams for patient treatment, most of them using Varian linear accelerators [15–23]. The flattening filter free technique became commercially available for Elekta linear accelerators in 2013, therefore only a few planning studies are available for Elekta up to now, most of them conducted in the treatment planning system Monaco for stereotactic treatments [24–27]. Only a few planning studies also consider the benefit of a potential reduction in peripheral dose [2, 25].

Since patients with spinal metastases suffer from enormous pain, a reduction in delivery time would mean less emotional strain for the patients as well as reduced risk for intrafractional motion [28]. A reduction in peripheral dose leads in general to a reduction in normal tissue complication probability. The purpose of the project presented here was therefore to investigate the benefit of flattening filter free beams in the re-irradiation of spinal column metastases considering plan quality, total delivery time and peripheral dose.

## Methods

### Patients

CT data of ten patients with spinal column metastases who had previously been treated with 3D-CRT were selected from our treatment database. These data had previously been used for another study with identical delineation of the planning target volume (PTV) and organs at risk (OAR) and dose prescription [29]. The PTV of the first course (pre-irradiation) consisted of the 1–5 thoracic vertebrae including the spinal cord. For this study it was assumed that the patients have an in-field recurrence and the whole vertebra region including the spinal canal was pretreated with a dose of 10 × 3 Gy in the first course. For the second course considered in this planning study, the CTV consists of one to five whole vertebrae including the vertebral body, the vertebral arch, the transverse processes and the spinous process. The spinal canal is excluded from the CTV and considered as organ at risk. The PTV is defined as CTV extended by a 3 mm margin in each direction excluding the spinal canal from this expansion. Volumes of the PTV ranged from 101 to 388 ccm, with a cranio-caudal extension ranging from 4 to 11.5 cm. Prescription for the second course is 6 × 4 Gy average dose to the PTV. To avoid myelopathy the dose to the spinal cord was restricted to 18 Gy due to the exposure in the first series.

The restriction to 18 Gy was calculated based on the risk score model of Nieder et al. [30, 31] who found no incidence of radiation myelopathy after a total biologically effective dose BED of 120 Gy_2_, if the interval between radiation courses was at least 6 months and the BED of each course was not higher than 98 Gy_2_. The α/β value for spinal cord was hereby assumed to be 2 Gy [30, 31]. The biologically effective dose is defined as BED = n ∙ d ∙ (1 + d/(α/β)) according to Fowler [32], where n is the number of fractions, d the dose per fraction and α and β the coefficients of the linear quadratic cell model. Since the BED of the first course considered in our study was 75 Gy_2_, the remaining maximum BED for the second course was 45 Gy_2_ corresponding to 6 × 3 Gy to assure a total maximum BED of 120 Gy_2_, i.e. a 0 % risk of radiation myelopathy. Details about the calculation are described in Groeger et al. [29].

### Linear accelerator and treatment planning system

Treatment planning is performed with Oncentra® External Beam v4.5 (Nucletron, an Elekta Company) for a Synergy linear accelerator with Agility™ head (Elekta AB, Stockholm, Sweden) and six MV photons with flattening filter (FF) or without (FFF). The FFF beams were energy-matched to the FF beams as it is common for Elekta accelerators [12, 13, 33]. The multi leaf collimator consists of 80 leaf pairs of 5 mm width at isocenter. The maximum nominal dose rate is 500 MU/min in FF Mode and 1700 MU/min in FFF mode. Beam profiles, depth doses and dose output were found to be stable for 4 MU and larger in both irradiation modes as also reported by Akino [14]. Verification of the linac model in Oncentra by collapsed cone dose calculations of percentage depth doses, profiles and output factors was within specifications of Oncentra®, i.e. 3 % of calibration dose in the dose plateau and 3 mm distance deviation to correct dose value in sharp dose gradients, for both FF and FFF. The accuracy of the collapsed cone dose calculation algorithm implemented in Oncentra has previously been reported to be at least as high for FFF beams as for FF beams [34].

### Treatment planning

In total four treatment plans were created for each patient, using two different treatment techniques IMRT and volumetric modulated arc therapy (VMAT) and two different irradiation modes with and without flattening filter. In the following the plans are referred to as IMRT FF, IMRT FFF, VMAT FF and VMAT FFF. The IMRT plans consist of nine equispaced beams, minimal segment size was 9 cm^2^, maximal number of segments allowed was 70. Minimal number of monitor units per segment is four due to the determined stability of the beam for 4 MU and higher. The VMAT plans consist of two full rotations with gantry spacing between two control points of 4°. Collimator angles ranged from 0 to 45° for both techniques. Identical dose volume objectives (DVO) and weights were used for optimization of all plans (Table 1). Suitable DVO and weights were determined creating plans in FF mode which met the goals and then transferred to the FFF plans. All plans were accepted for treatment by a specialized radiation oncologist.

**Table 1:** Dose Volume Objectives (DVO)

Organ	Type	DVO	relative weight
PTV	target	uniform dose 24.0 Gy	7000
		minimum dose 23.5 Gy	7000
		maximum dose 24.5 Gy	7000
spinal canal	organ at risk	maximum dose 18.0 Gy	750
spinal cord	organ at risk	maximum dose 16.0 Gy	1000
normal tissue	organ at risk	maximum dose 24.5 Gy	5000
		surrounding dose fall off from 24.0 to 4.8 Gy in 5.0 cm	5000

Identical dose volume objectives (DVO) and weights were used for optimization of all plans

### Dosimetry

**Fig. 1:**
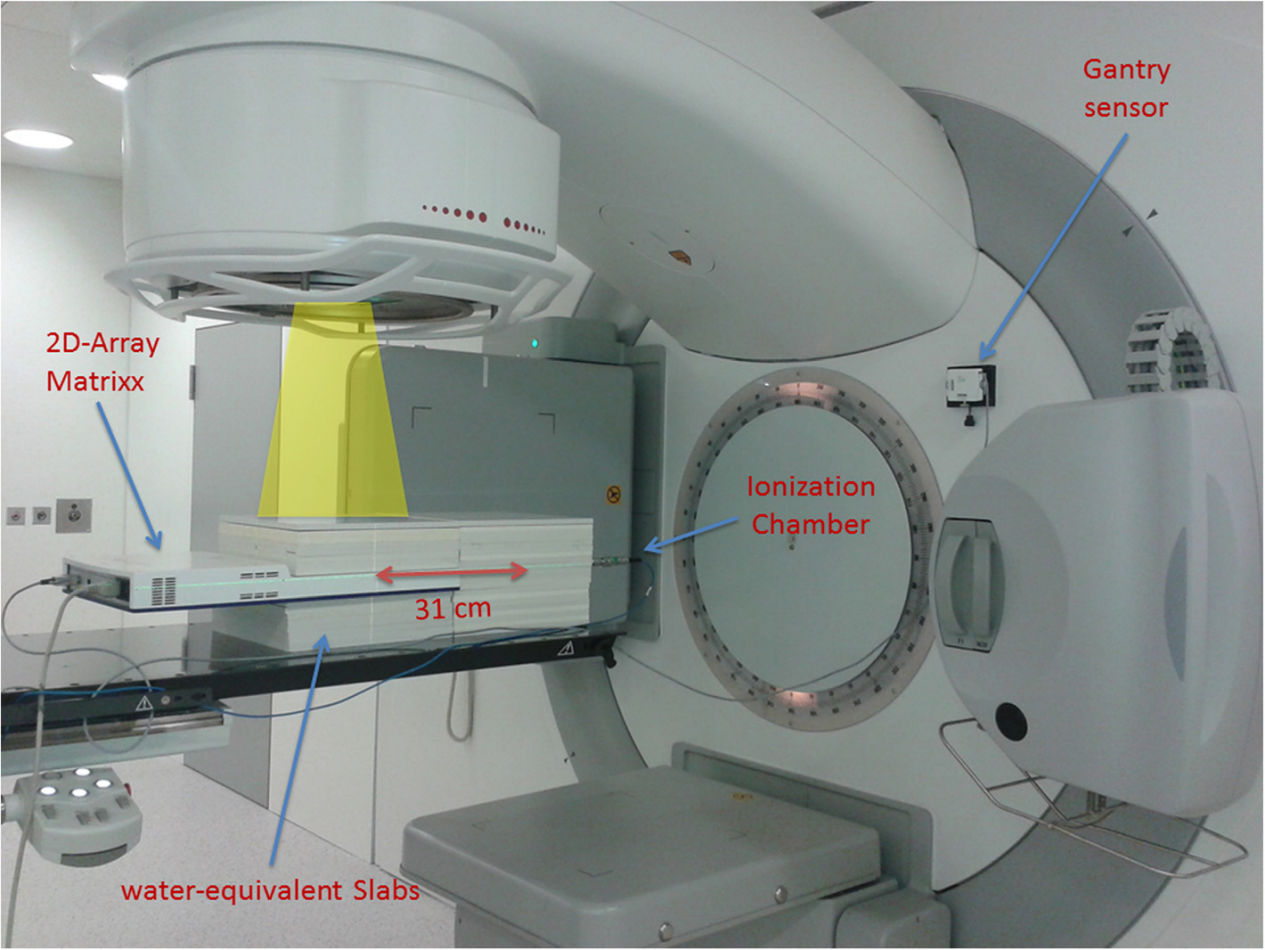
Measurement setup at the linac. The 2D-ionisationchamber-array MatriXX Evolution^TM^ was set up between slabs of RW3 and centered at the isocenter of the linac. The setup was extended cranially by additional slabs of RW3 of the same height with a 0.3 ccm ionisation chamber positioned 31 cm cranially of the isocenter

For verification all 40 plans were transferred to a CT scan of the MatriXX Evolution™ 2D-ionisationchamber-array (IBA Dosimetry, Schwarzenbruck, Germany) set up in between slabs of a RW3 phantom (PTW, Freiburg, Germany) for measurement in a coronal plane [35, 36]. The 2D-ionisationchamber-array MatriXX Evolution™ consists of 1020 vented pixel ionisationchambers arranged in a square of 24.4 cm × 24.4 cm with a center-to-center distance of 0.76 cm, the chamber size is 0.45 cm diameter and 0.5 cm height, the active volume is 0.08 cm^3^, and RW3 is used as backscatter material. RW3 consists of white polystyrene and is dosimetrically water-equivalent for photons in the range of ^60^Co to 25 MV. The isocenter was placed such that the measurement plane intersected both the PTV and the spinal cord in order to verify the dose to the PTV and the dose to the organ at risk at the same time. This is possible considering the diameter of the spinal cord of around 1.5 cm, the chamber distance of 0.76 cm and the active chamber volume of 0.08 cm^3^. It should be mentioned, however, that the measurement of very steep gradients between PTV and spinal cord is limited by the chamber distance. The dose in the measurement plane was calculated with a dose grid resolution of 0.15 cm, all other parameters were kept identical to the patient plan. The plans were then delivered to the phantom. Measurements were corrected for angular dependencies and couch attenuation in the steering and evaluation software OmniPro I’mRT v.1.7a (IBA Dosimetry, Schwarzenbruck, Germany). In addition a point dose measurement was performed in the same coronal plane but 31 cm cranial of the isocenter using a 0.3 ccm PTW ionization chamber to assess peripheral dose. The dose at the point of measurement was calculated in Oncentra on the CT scan of the phantom. The complete measurement setup is shown in Fig. 1.

### Efficiency

Delivery times were measured from first beam on to last beam off to assess the achievable reduction in delivery time. In addition the number of required monitor units (MU) per Gray was compared.

### Evaluation

Plan quality was assessed by analysis of the dose volume histogram (DVH) with respect to target coverage, dose homogeneity and conformity, dose to the spinal cord and normal tissue. Target coverage was represented by the volume of the PTV covered by 95 % of the prescription dose (V_95%_). The homogeneity index was defined as HI := (D_1%_ - D_99%_)/D_50%_, the conformity index according to Paddick et al. [37] as CI := V_95%_^2^/(TV ⋅ PIV). Here TV means the volume of the PTV, PIV the total volume covered by 95 % of the prescription dose. To assess the sparing of the spinal cord as described in the treatment goals, the volume V_75%_ covered by 75 % of the prescription dose i.e. 18 Gy was recorded. The value of 18 Gy was calculated as residual tolerance dose due to the exposure in the first series. According to ICRU report 50 “a significant tissue volume must be irradiated for the dose level to be reported as maximum” [38]. ICRU report 80 suggests that D_2%_ may be an appropriate value if the whole structure is delineated [39], which corresponds to a volume of about 1 ccm which is also commonly used in the literature [40]. The median dose D_50%_ of the normal tissue, which is defined as the patient body excluding the PTV, is listed as a measure of low dose in the periphery.

For evaluation of plan verifications gamma indices as defined by Low et al. [41] were calculated with a dose tolerance of 3 % of the maximum dose and 3 mm distance to agreement. Dose calculations are considered acceptable if at least 95 % of the pixels with a dose value of ≥ 10 % of the maximum dose have a gamma value ≤ 1 as recommended by the AAPM TG119 [42, 43]. Ionchamber point dose measurements and corresponding calculations 31 cm cranial of the isocenter were compared for the two irradiation modes to assess peripheral dose.

The Wilcoxon test implemented in IBM SPSS® Statistics 23.0 (IBM Corporation) was used for statistical analysis with a significance level of 0.05 of a) the FF mode versus the FFF mode separated by the planning techniques IMRT and VMAT and b) IMRT versus VMAT separated by the irradiation mode FF and FFF. For the assessment of peripheral dose and delivery time, the Wilcoxon test was also performed for all plans in FF mode versus FFF mode.

## Results

Since the main subject of the study was the comparison of the two irradiation modes FF and FFF, details about statistical significance are listed in the tables for these Wilcoxon tests. Differences between IMRT and VMAT are mentioned in the text but not listed in detail in the tables for the sake of clarity.

### Plan quality

**Fig. 2:**
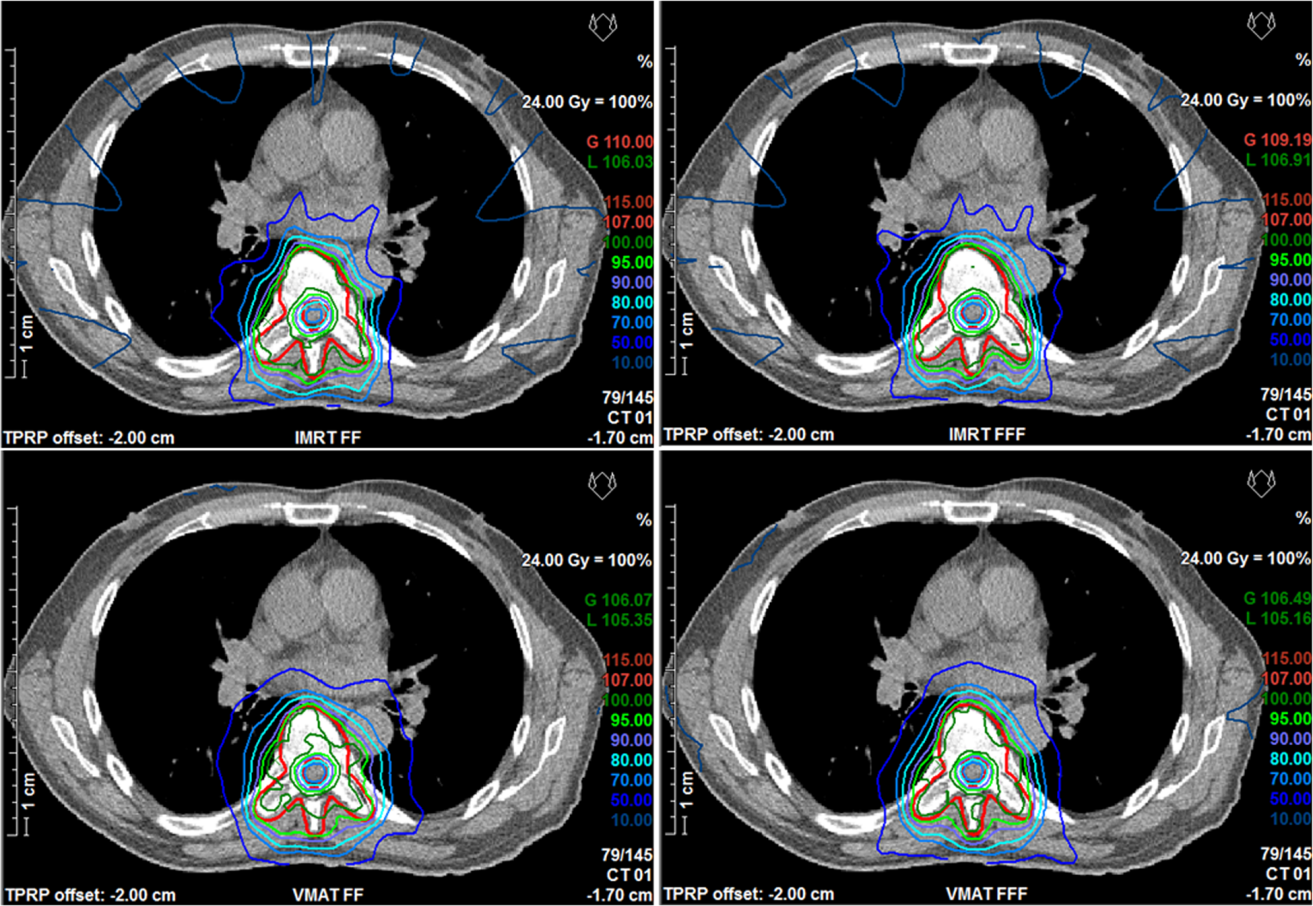
Comparison of dose distributions. Comparison of dose distributions in one transversal slice for a representative case. Top IMRT, bottom VMAT, left FF, right FFF

**Fig. 3:**
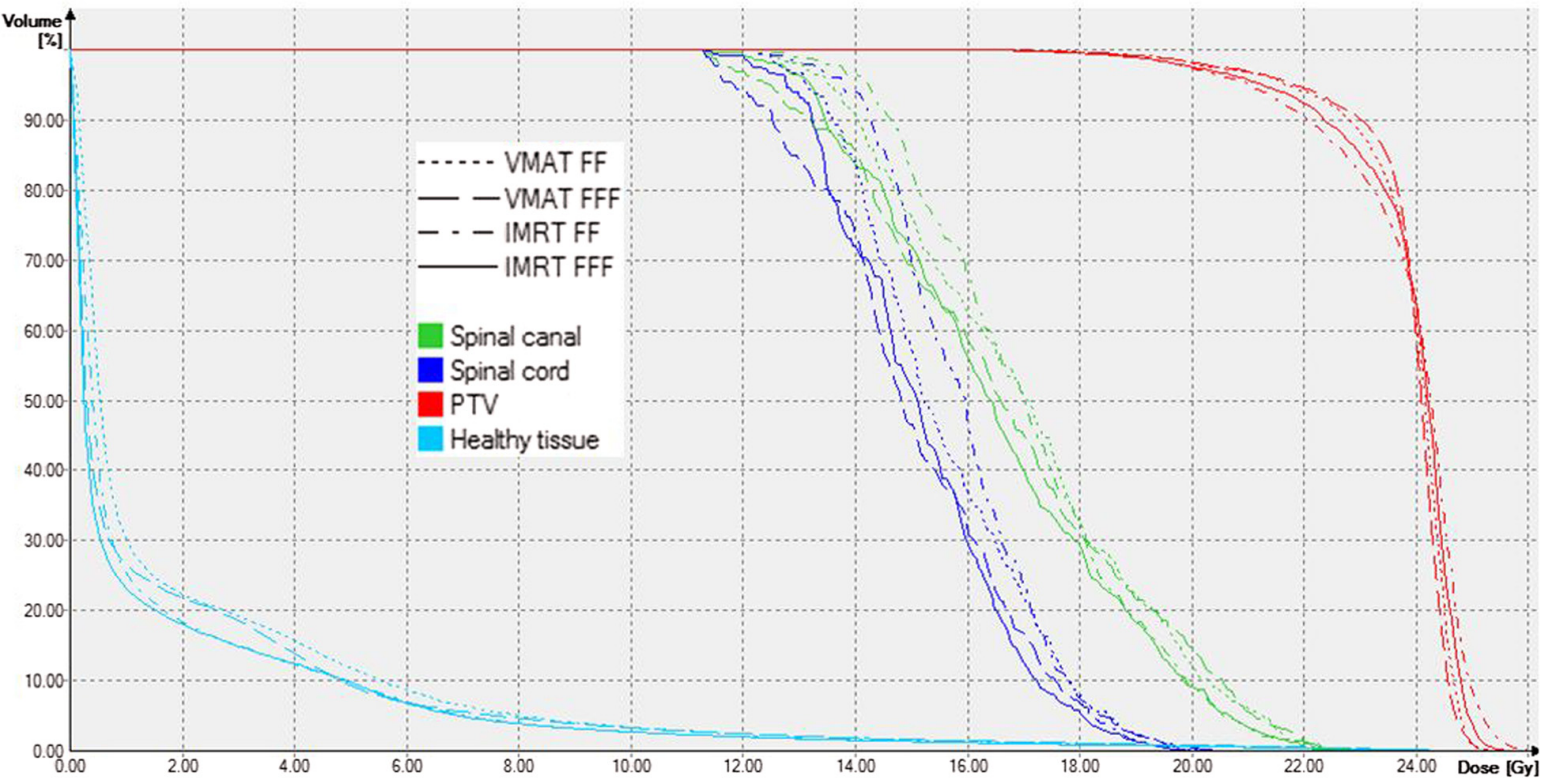
Comparison of dose volume histograms. Comparison of dose volume histograms for the case of Fig. 2

Analysis of the dose volume parameters listed in detail in Table 2 shows slightly higher plan quality for both IMRT and VMAT, if flattening filter free beams were used: the volume of the spinal cord receiving the allowed maximum dose as well as the dose to the normal tissue could be significantly reduced keeping target coverage, homogeneity and conformity at the same level. The volume of the spinal cord receiving 18 Gy was kept well below the clinically relevant volume of 1 ccm in all cases if FFF mode was used but exceeded 1 ccm in 50 % of the cases for FF mode, with a maximum volume of 2.2 ccm. Comparison of VMAT versus IMRT also showed significant differences in target coverage and homogeneity as well as sparing of the spinal cord and normal tissue. Best target coverage and homogeneity were achieved with VMAT FFF, whereas lowest doses to the spinal cord and normal tissue were achieved with IMRT FFF. A comparison of dose distributions and dose volume histograms is shown in Figs. 2 and 3 for a typical case.

**Table 2:** Comparison of plan quality

		IMRT			VMAT		
Parameter		FF	FFF	*p*	FF	FFF	*p*
PTV	V_95%_ (%)	86.4 ± 1.7	**88.4 ± 1.7**	<0.01	91.0 ± 1.5	91.7 ± 1.3	0.14
	HI	0.27 ± 0.02	0.26 ± 0.02	0.06	0.23 ± 0.02	0.23 ± 0.02	0.96
	CI	0.65 ± 0.04	0.66 ± 0.04	0.17	0.65 ± 0.04	0.66 ± 0.04	0.51
Spinal Cord	D_50%_	15.9 ± 0.6	**15.1 ± 0.5**	<0.01	15.6 ± 0.3	**15.0 ± 0.4**	0.01
	V_75%_ (ccm)	0.9 ± 0.3	**0.5 ± 0.1**	<0.01	1.0 ± 0.5	**0.7 ± 0.2**	0.01
Normal Tissue	D_50%_	0.8 ± 0.4	**0.6 ± 0.4**	<0.01	1.1 ± 0.7	**0.8 ± 0.6**	<0.01

Mean values and standard deviation of the dose volume parameters for the four different planning techniques averaged over all patients. Dose values are given in Gy. *P*-values for comparison of FF and FFF are calculated separately for IMRT and VMAT. Bold values indicate significantly superior values

### Dosimetry

All 40 plans passed the gamma evaluation. Passing rates were similar for both techniques and showed no significant difference between FF and FFF or IMRT and VMAT. Details are listed in Table 3.

**Table 3:** Comparison of plan delivery and evaluation of dosimetry

	IMRT			VMAT		
Parameter	FF	FFF	*p*	FF	FFF	*p*
Delivery time (s)	557 ± 41	**438 ± 23**	<0.01	216 ± 15	**163 ± 5**	<0.01
Monitor units per Gy	**381 ± 25**	448 ± 43	<0.01	**391 ± 29**	429 ± 37	0.02
Measured peripheral dose (mGy)	8.6 ± 1.4	**5.8 ± 1.2**	<0.01	7.0 ± 1.0	**4.8 ± 1.0**	<0.01
Calculated peripheral dose (mGy)	4.7 ± 1.3	4.6 ± 1.5	0.51	5.7 ± 1.4	5.6 ± 1.4	0.24
Passing rate of γ (%)	97.6 ± 1.1	97.4 ± 1.2	0.51	98.1 ± 1.1	97.9 ± 1.0	0.37

Mean values and standard deviation of delivery time, monitor units, measured and calculated peripheral doses and result of the gamma evaluation for the four different planning techniques averaged over all ten patients. *P*-values for comparison of FF and FFF are calculated separately for IMRT and VMAT. Bold values indicate significantly superior values

Out-of-field point doses measured at 31 cm from the isocenter were significantly reduced for FFF as compared to FF. Averaged over all plans a dose reduction of 33.2 % ± 5.4 % of the local dose was achieved. The lowest out-of-field point dose was found for VMAT FFF, which was significantly lower than for IMRT FFF (*p* < 0.01). Calculation of the peripheral dose at the point of measurement in the phantom in Oncentra showed no significant differences between the FF and FFF. Comparison of calculated versus measured peripheral doses showed a mean local dose deviation of - 33.6 % ± 16.6 % for flat beams and −2.0 % ± 29.4 % for FFF beams. Details are listed in Table 3.

### Efficiency

FFF Plans required significantly more MU per fraction dose for both IMRT and VMAT, whereas no significant difference could be found between IMRT and VMAT in both irradiation modes. Details are listed in Table 3.

Delivery time was significantly reduced for FFF beams in both treatment techniques. Averaged over all patients the delivery time was reduced from 557 to 438 s for IMRT and 216 s to 163 s for VMAT, which corresponds to a reduction of 21 % ± 4 % for IMRT and 24 % ± 5 % for VMAT. The lowest delivery time was found for VMAT FFF, which was significantly lower than for IMRT FFF (*p* < 0.01). Details about delivery times are listed in Table 3.

## Discussion

The aim of this study was to investigate the potential of the flattening filter free (FFF) mode of a linear accelerator for intensity modulated radiation therapy (IMRT) and volumetric modulated arc therapy (VMAT) for patients with in-field recurrence of vertebral metastases. The data show significant advantages for FFF beams as compared to FF beams in terms of plan quality as well as delivery time and peripheral dose: FFF offered a significant improvement in the sparing of normal tissue and the spinal cord, which could be of importance, minimizing the risk of radiation myelopathy, keeping target coverage and homogeneity at the same level. According to the literature [31], the risk for radiation myelopathy is 0 % if the BED of each series is ≤ 98 Gy_2,_ the interval between the series is at least 6 months, and the total BED of all series is ≤ 120 Gy_2_. The risk increases to 3 % for an upper limit of the total BED of 135.5 Gy_2_. In the scenario presented here this means that the dose to the spinal cord has to be restricted to 18 Gy to assure a 0 % risk of myelopathy. The volume of the spinal cord receiving 18 Gy was kept well below the clinically relevant volume of 1 ccm in all cases if FFF mode was used, but exceeded 1 ccm in 50 % of the cases, when FF mode was used. The reason for the better sparing of the spinal cord may be found in the shape of the dose profiles for beams with the central part blocked: For the peak formed profile of the FFF beams the gradient towards the blocked center is slightly steeper than of FF beams. The lower dose to the normal tissue in the periphery can be explained by reduced head scatter if the flattening filter is removed. In spite of increased MU in the FFF mode, delivery time and peripheral dose exposure are reduced, which means less strain for the patient and possibly reduced risk of normal tissue complications. When compared to IMRT FF, VMAT FFF offered a reduction of 71 % in delivery time and 45 % peripheral dose. According to Ma et al. [28] the critical time to keep target motion within 1 mm of translation or 1° of rotational deviation is 5.9 min (354 s) when patients are immobilized in a vacuum cushion. This was achieved in all cases of our study for VMAT only. In summary VMAT FFF may therefore be considered the preferable treatment option for radiotherapy of spinal column metastases which the combination of Elekta Synergy linacs with Agility™ head and the treatment planning system Oncentra® External Beam v4.5.

Concerning plan quality there is a broad variety in the studies published up to now: For small targets with low modulation, as they are common in stereotactic treatments, plan quality was mostly found to be comparable [19, 26]. For larger targets, one group reported similar target coverage at significantly reduced doses to organs at risk for FFF beams [15, 21] which is in concordance with our findings. Other studies found superior plan quality for flat beams [20, 44, 45]. The only planning study dealing with irradiation of vertebrae with FFF beams has to our knowledge been published by Ong et al. for RapidArc plans created in the Eclipse planning system for Varian linear accelerators [23]. In their study different fractionation schemes of single doses ranging from 9 to 16 Gy were used and plans were normalized with respect to acceptable dose to the spinal cord. Ong et al. found slightly increased target dose for FFF as compared to FF, which is in concordance with the results of our study presented here. However, photon energies used in their study differed between FF (6 MV) and FFF (10 MV), which might bias the results, whereas we used the same energy for both irradiation modes in our study. Validation of the dose calculation by measurements in the study of Ong et al. showed good agreement for a region receiving more than 20 % of the prescription dose, evaluation of peripheral low dose was not performed. Reduction in delivery time was more pronounced in the study of Ong et al., with a higher mean for FF (402 s vs 216 s) and a comparable mean for FFF (168 s versus 163 s), since the benefit of higher dose rates is more pronounced for higher fraction doses as they are used in the study of Ong.

Reduction in out-of-field dose has been reported previously in the context of Monte Carlo simulations of FFF beams for both Elekta [13] and Varian [46]. The authors emphasize, however, that a reduction of peripheral dose in patient treatments “may not always be achievable” due to dependence on many parameters [46]. Most of the planning studies published up to now did not take peripheral dose measurements into account. Spruijt et al. [20] performed a planning study for breast cancer and created one phantom case for out-of-field dose measurements 0.3 cm to 3.1 cm from the field edge. They found an average reduction in out-of-field dose of 10 %. Comparison to dose calculations in this region showed, however, an underestimation by the treatment planning system Eclipse of 26 % to 85 % as compared to measurements. Absolute dose values are not reported. Because of the uncertainties in the dose calculation the authors abandoned evaluation of dose volume parameters of the contralateral breast in their planning study. Kragl et al. [2] measured peripheral dose for four sample cases, one 7-field phantom plan, one lung SBRT, one prostate IMRT and one head and neck IMRT case and found a substantial local dose reduction at around 20 cm from the field edge. Because only one case per technique was considered, no conclusion with statistical significance could be drawn. The results of our study evaluated for a larger number of cases support their findings with statistical evidence. As Kragl et al. pointed out, peripheral doses are difficult to calculate correctly with a high accuracy, and should therefore be determined by measurements or Monte Carlo simulations. Therefore they did not compare the peripheral dose measurements with calculations. Comparison of calculated versus measured peripheral doses in our study showed significantly higher agreement between calculated and measured peripheral doses for FFF beams than for FF beams. The calculation in Oncentra did not reveal the actual reduction of peripheral dose which is possible to achieve by the use of FFF due to a systematic underestimation of the peripheral dose for FF beams, which was not observed for FFF beams. Thus, the advantage of FFF determined in the calculated DVH of the normal tissue is even underestimated by the dose calculation in Oncentra.

## Conclusions

For the combination of an Elekta Synergy linac with Agility™ head and the treatment planning system Oncentra® External Beam v4.5 the use of flattening filter free beams in re-irradiation of spinal column metastases allows better sparing of the spinal cord, minimizing the risk of radiation myelopathy, without compromising target coverage and homogeneity for both IMRT and VMAT. Delivery time is significantly lower as compared to flat beams, which may reduce intrafractional movement and emotional strain to the patient. When compared to IMRT FF, VMAT FFF offered a reduction of 71 % in delivery time and 45 % in peripheral dose. For radiotherapy of spinal column metastases VMAT FFF may therefore be considered the preferable treatment option for the combination of Elekta Synergy linacs with Agility™ head and the treatment planning system Oncentra® External Beam v4.5.

## Abbreviations

CI: conformity index
ccm: cubic centimeter
DVH: dose volume histogram
DVO: dose volume objective
D_x_: dose to volume x of the structure
FF: flattening filter
FFF: flattening filter free
HI: homogeneity index
IMRT: intensity modulated radiation therapy
min: minutes
PTV: planning target volume
PIV: total volume of the 95 % isodose
s: seconds
TV: volume of the PTV
VMAT: volumetric modulated arc therapy
V_x%_: volume covered by x% of the prescription dose
